# Matrix Metalloproteinases MMP-2 and MMP-9 Occupy a New Role in Severe Preeclampsia

**DOI:** 10.1155/2020/8369645

**Published:** 2020-12-16

**Authors:** Elena Timokhina, Alexander Strizhakov, Sapiyat Ibragimova, Evgeny Gitel, Irina Ignatko, Vera Belousova, Nikoleta Zafiridi

**Affiliations:** ^1^Department of Obstetrics, Gynecology and Perinatology, I.M. Sechenov First Moscow State Medical University (Sechenov University), B. Pirogovskaya 2-4, 119991 Moscow, Russia; ^2^Centralized Laboratory Diagnostic Service, I.M. Sechenov First Moscow State Medical University (Sechenov University), B. Pirogovskaya 2-4, 119991 Moscow, Russia

## Abstract

**Introduction:**

Preeclampsia (PE) is a life-threatening condition for the mother, the fetus, and the newborn. Matrix metalloproteinases (MMP) participate in the two primary stages of PE: remodeling of blood vessels at the stage of placental formation and the development of hypertension due to damage to the basement membrane of blood vessels. The object of the present study was to reveal the role of MMP-2 and MMP-9 in the development of severe preeclampsia.

**Materials and Methods:**

We conducted a retrospective study that included 92 pregnant women at a gestational age of 26-38 weeks, of which the principal group consisted of 61 patients with severe PE. We divided the principal group into two subgroups: the first subgroup was designated the severe early-onset preeclampsia (EO-PE) group and consisted of 30 pregnant women. The second group was designated the severe late-onset preeclampsia (LO-PE) group, comprising 31 patients. We determined the plasma concentrations of MMPs 2 and 9 in the groups with an ELISA.

**Results:**

In the group of PE patients with both EO-PE and LO-PE, the level of MMP-2 was significantly higher compared to the women undergoing normal pregnancy; and we observed no significant differences when we compared the levels of MMP-2 in the subgroups with EO-PE and LO-PE. Analysis of the concentrations of MMP-9 in EO-PE and LO-PE subgroups revealed attenuated levels of MMP-9 in both groups relative to the control group. We also noted a diminished level of MMP-9 in the EO-PE group compared to the LO-PE group.

**Conclusions:**

The significantly increased levels of MMP-2 in women—both in the EO-PE and LO severe PE subgroups—explain the participation of this enzyme in endothelial dysfunction in the second stage of severe PE. A diminution in MMP-9 in the EO-PE group confirmed the participation of MMP-9 in the process of spiral artery transformation.

## 1. Introduction

Preeclampsia (PE) is a life-threatening condition for the mother, fetus, and newborn. Worldwide, it is diagnosed annually in 10 million women, which is between 3% and 8% of all pregnancies. By conservative estimates, these disorders are responsible for 76,000 maternal and 500,000 infant deaths each year. PE induces multiple-organ damage to a woman's organs and systems in the second half of pregnancy (after 20 weeks) due to systemic endothelial dysfunction. The outcome of severe PE is cerebral edema, the development of seizures (eclampsia), and hepatic, renal, and cardiovascular failure. PE is therefore a prominent cause of death of both mother and fetus [2–6]. The primary element in the development of PE is a disorder of placental formation, a process that occurs in the first 13 weeks of pregnancy, i.e., long before the first clinical manifestations of the symptoms [2–5]. The leading cause of PE is incomplete trophoblast invasion and remodeling of the spiral arteries, which lead to unstable perfusion of the intervillous space, ischemic and reperfusion damage of the placental tissue, and systemic oxidative stress. This complex process is carried out due to the migration of extravillous trophoblast deep into the endometrium—termed cytotrophoblastic invasion [2–4]. The reason for the incomplete reconstruction of the spiral arteries leading to PE is the insufficient activity of the lysing enzymes known as matrix metalloproteinases (MMPs).

MMPs are extracellular proteinases that play an important role in physiologic and pathologic processes: embryogenesis, implantation, placental formation, neoangiogenesis, and tumor transformation [1–4]. The first MMP was identified in 1962 as a protease responsible for the degradation of fibrillar collagen in tadpole tails during metamorphosis and was therefore called interstitial collagenase. After the identification of a similar collagenase in human skin, this protease was renamed MMP-1 [1]. MMPs are secreted by various cell types, including neutrophils, fibroblasts, epithelial cells, macrophages, vascular endothelial smooth muscle cells, and osteoblasts [1]. With ongoing development during pregnancy, MMPs take part in the processes of blastocyst implantation, gestational transformation of the spiral arteries, and the formation of the placenta [2–5]. Successful implantation occurs due to the ability of trophoblast cells to cleave the extracellular matrix components, in which MMPs are directly involved [2, 3, 7]. Changing the concentration and activity of metalloproteinases is therefore the subject of close attention of PE researchers [5].

The second stage of PE is endothelial damage with the development of arterial hypertension (AH) and multiorgan damage due to the initiation of oxidative stress within the placenta, releasing pro- and antiangiogenic factors, with MMPs also referred to as antiangiogenic factors. MMPs damage the endothelium, cause systemic endothelial dysfunction, lead to AH, and can result in multiple-organ failure [3]. As we currently understand it, the participation of MMPs in the development of hypertension is due to their influence on the vasoactive properties and architectonics of the vessel walls, as MMPs take part in the synthesis of the vasoconstrictor endothelin [8].

Another potential mechanism underlying the formation of hypertension may be due to the direct destructive effect of MMPs on the proteins of the extracellular matrix and basement membranes by the destruction of collagen-1 [3]. Two members of the MMP family, MMP-2 and MMP-9, are involved in the processes of cytotrophoblastic invasion of the mother's vasculature and in the remodeling of the placental and uterine arteries [3, 4]. MMP-2 and MMP-9 also play roles in the remodeling of blood vessels at the stage of placental formation and in the development of hypertension due to damage to basement membrane vasculature. Thus, MMPs are involved in both the first and second stages of PE [2, 4].

The current classification scheme differentiates PE into two variants [5]. Early-onset PE (EO-PE) develops to and requires delivery at 34 weeks of gestation. The cause of EO-PE is a placental factor that induces insufficient reconstruction of the spiral arteries during the second wave of trophoblast invasion, decreased perfusion of the intervillous space, and placental ischemia with the development of oxidative stress. Late-onset PE (LO-PE) appears after 34 weeks of pregnancy as a metabolic syndrome and is not associated with the formation and functioning of the placenta in the second half of pregnancy. Rather, it is manifested by increased blood pressure, proteinuria, and edema. Studies on the role of MMPs in PE are few and ambiguous, and therefore, in the present study, we aimed to study the role of MMP types 2 and 9 in the development of severe PE with an early vs. late onset.

## 2. Materials and Methods

We conducted a retrospective study in which we enrolled 92 pregnant women with severe PE at a gestational age of 26–38 weeks. Informed consent was obtained from each woman to participate in the present study, and the study protocol was approved by the Sechenov University Ethics Committee (No. 10-17 of November 16, 2017). The main group consisted of 61 patients with severe PE. The criteria for inclusion in the main group were the presence of one and/or several criteria for PE with severe features.

Severe features of preeclampsia include any of these findings:

- Systolic blood pressure of 160 mmHg or higher or a diastolic blood pressure of 110 mmHg or higher on two occasions at least 4 hours apart while the patient was on bed rest (unless antihypertensive therapy was initiated prior to this time)
- Thrombocytopenia (platelet count less than 100,000/microliter)
- Impaired liver function as indicated by abnormally elevated blood concentrations of liver enzymes (to twice the normal concentration), severe persistent right upper quadrant or epigastric pain unresponsive to medication and not accounted for by alternative diagnoses, or both
- Progressive renal insufficiency (a serum creatinine concentration greater than 1.1 mg/dl or a doubling of the serum creatinine concentration in the absence of other renal disease)
- Pulmonary edema
- New-onset cerebral or visual disturbances

In order to study the role of MMPs in the onset and progression of PE in the main group, we differentiated the main group into two subgroups. The first subgroup we designated severe EO-PE and comprised 30 pregnant women. The second subgroup we defined as severe LO-PE and was composed of 31 patients. All patients were delivered by cesarean section due to severe PE and/or fetal distress. Criteria for exclusion from the study were patient failure, premature rupture of membranes, chorioamnionitis, diabetes mellitus, autoimmune disorders, and severe heart, kidney, and liver diseases before pregnancy. The control group consisted of 31 patients with uncomplicated pregnancy.

The levels of MMP-2 and MMP-9 were determined in plasma by enzyme-linked immunosorbent assay (ELISA) according to the manufacturer's instructions (Cloud-Clone Corp. Houston, USA). The plasma samples were taken at the moment of diagnosis of severe PE.

We performed statistical analysis with the SPSS program using Student's *t*-test.

## 3. Results

The general clinical characteristics and obstetric history of the study population are shown in Table 1.

We found no statistically significant differences among the groups with respect to the average age of the mother. Multiparous patients prevailed in the main group; and therefore, the control group was selected as a comparator. The study groups did not differ by parity. BMI was higher in the main group compared with the control but did not differ between the severe EO-PE and LO-PE groups. We noted a significantly higher level of SBP and DBP in the 2 study groups with PE compared with uncomplicated pregnancies. The average gestational age at delivery in the EO severe PE subgroup was 30.27 ± 2.90 weeks, and in the LO severe PE subgroup, it was 37.07 ± 1.73 weeks—which was comparable to the women with normal, uncomplicated pregnancies (38 ± 3.63).

### 3.1. MMP-2

**Figure 1:**
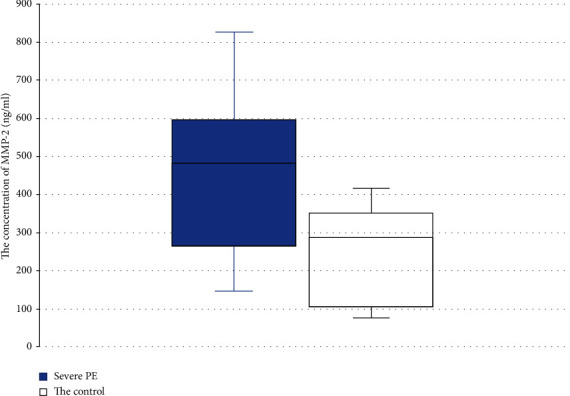
Concentration of MMP-2 in the severe PE group vs. normal pregnancy. Box plot showing the median (bold horizontal line), interquartile range (box), and total range (whiskers).

Our study revealed that in the main group (women with PE), the concentration of MMP-2 was 499 ± 216 ng/ml, significantly higher than in the control group −257 ± 123 pg/ml (Figure 1).

We analyzed the levels of MMP-2 in the two subgroups of the main group (early- and late-onset PE) and found that in both subgroups of patients with PE, the levels of MMP-2 were significantly higher than in healthy pregnant women in the control group at the same gestational age.  Table 2 shows the levels of metalloproteinases in the plasma samples of the women with EO-PE and LO severe PE and the control group.

**Figure 2:**
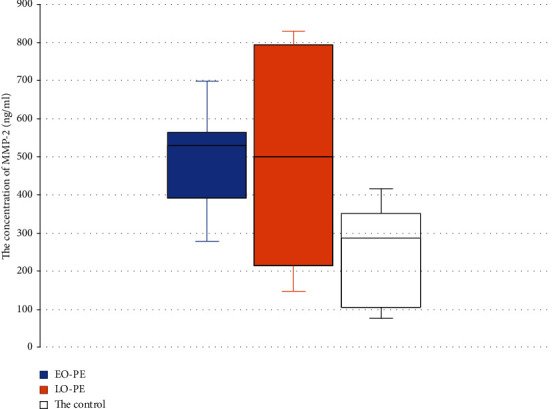
Comparison of the levels of MMP-2 in the severe EO-PE and LO-PE subgroups and the control group. We did not observe a difference in the levels of MMP-2 in the EO-PE and LO-PE subgroups. Box plot showing the median (bold horizontal line), interquartile range (box), and total range (whiskers).

In the EO-PE subgroup, the level of MMP-2 was 508 ± 137 ng/ml, and in the late-onset PE subgroup, it was 491 ± 275 ng/ml (NS between subgroups), with both indicators differing significantly from the concentration of MMP-2 in the control group −257 ± 123 ng/ml (Figure 2).

### 3.2. MMP-9

**Figure 3:**
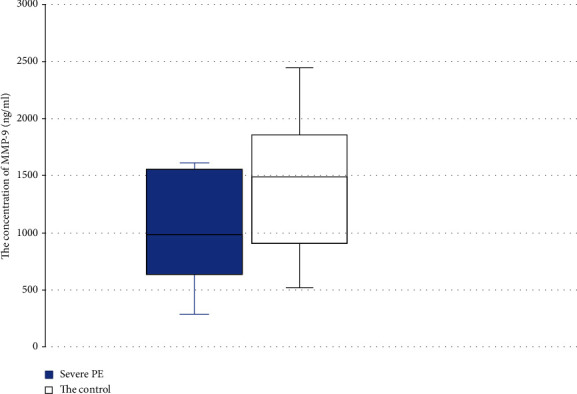
Concentration of MMP-9 in the groups with severe PE versus normal pregnancy. Box plot showing the median (bold horizontal line), interquartile range (box), and total range (whiskers).

Although the levels of MMP-9 showed a tendency to be lower in the PE group relative to the control group, this was not statistically significant (970 ± 439 ng/ml vs. 1391 ± 599 ng/ml, respectively) (Figure 3).

**Figure 4:**
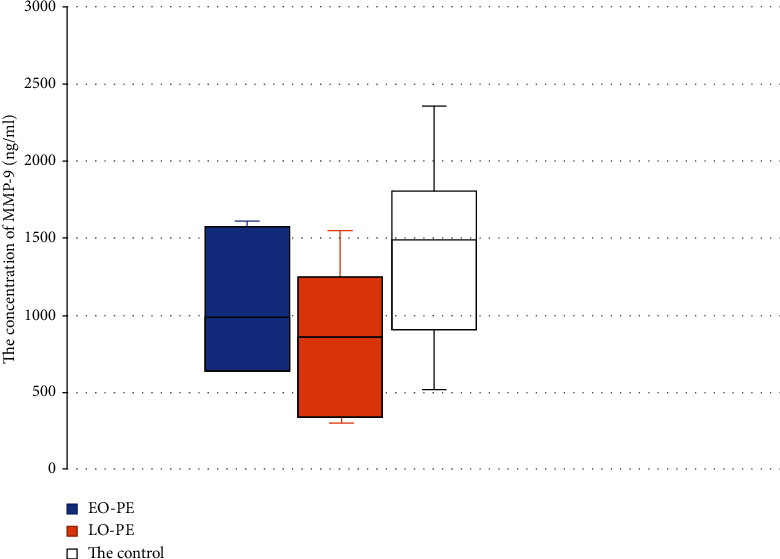
Comparison of the level of MMP-9 in the subgroups of EO-PE and LO-PE with the control group. Box plot showing the median (bold horizontal line), interquartile range (box), and total range (whiskers).

A detailed analysis of the concentration of MMP-9 in the EO-PE and LO-PE subgroups showed that the levels of MMP-9 were lower in both subgroups relative to controls (Figure 4). In the EO-PE subgroup, the MMP-9 level was 1116 ± 389 ng/ml—which was significantly lower than in the control group (1391 ± 599 ng/ml). In the group of LO-PE, the level of MMP-9 was also lower than in the control group (834 ± 451 ng/ml vs. 1391 ± 599 ng/ml, respectively) (Figure 4).

When we compared the concentration of MMP-9 between the EO-PE and LO-PE subgroups, we noted a lower level with late-onset PE compared with early-onset PE (1116 ± 389 ng/ml vs. 834 ± 451 ng/ml, respectively) (*p* > 0.05).

## 4. Discussion

According to conventional medical wisdom, the first stage of PE is characterized as a disorder in the formation of the placenta. The process of placental formation and development entails a complex series of steps that involve trophoblast invasion, endothelial proliferation, degradation of the extracellular matrix so as to allow invasion by proliferating endothelial cells into the underlying tissues, and neoangiogenesis. And several researchers currently express the view that MMPs are involved in the above processes. A role for MMPs in PE refers to the second stage of this complicated process, which includes endothelial damage and impaired vasodilator/vasoconstrictive activities.

In the current study, we attempted to elucidate a potential role for MMPs in the genesis of severe PE and found that in the group of patients with both EO-PE and LO-PE, the levels of MMP-2 were significantly higher compared to the controls. We evaluated the concentrations of MMPs in patients with symptoms of PE and confirmed a role for MMP-2 in the development of endothelial dysfunction in this specific complication of pregnancy—results that are mirrored by the similar data of Laskowska [4]. In our study, we conversely found a decrement in the level of MMP-9 in pregnant women with PE, which is a metalloproteinase that is actively involved in remodeling of the spiral arteries at the first stage of PE development. The reduced levels of this enzyme in the plasma of pregnant women with PE thus reflect defective remodeling of the spiral arteries already at the first stage. We additionally noted a more significant decrease in MMP-9 in the group of pregnant women with EO-PE. Today, this form of PE is assessed as a true placenta-associated complication; i.e., its development is associated with defective angiogenesis during the formation of the placenta. Therefore, a significant decline in MMP-9 in the group with EO-PE confirms the participation of this enzyme in the process of transformation of the spiral arteries. Intriguingly, we found controversial data in the literature regarding the role of MMPs in the development of PE.

Although Montagnana et al. [6] also found high levels of MMP-2 and low levels of MMP-9 in serum in women with PE, these authors noted higher levels of MMP-2 in women with PE compared with healthy pregnant and nonpregnant women. Narumiya et al. showed results similar to ours, demonstrating an increased level of MMP-2 and a low level of MMP-9 in women with PE [7]. Gerlach et al. [9] did not recommend measuring MMPs in serum, because the level of MMP-9 in serum may be higher compared with levels observed in plasma, possibly due to the additional release of this MMP by platelets and leukocytes. Palei et al. [10] only observed augmented MMP-9 in pregnant women with gestational arterial hypertension and not with PE. According to their data, there were no significant differences in the levels of MMP-2 in either PE or gestational hypertension compared with control. Additionally, Reister et al. [11] surmised that MMP-2 reflected endothelial dysfunction during pregnancy complicated by PE and can also lead to the abnormal activity of vasoactive peptides and subsequently increased vasoconstriction.

The identification of a significantly elevated level of MMP-2 during pregnancy complicated by PE is potentially important, as it enables targeted therapy in PE by using tissue inhibitors of MMP (TIMP-2). This inhibitor binds MMPs in plasma and thereby circumvents damage to the endothelium and averts the clinical manifestations of PE. Studies on blocking MMPs have only recently begun on experimental animal models, and results have not yet been obtained but are anticipated.

Regarding the role of MMP-9 in the genesis of PE, we attribute its participation in angiogenesis to the formation of the placenta; MMP-9 is even referred to as a “trigger of angiogenesis” [6, 9]. Thus, the reduced levels of MMP-9 we and others have observed [6, 7, 10] can be explained in consonance with this point of view. The level of MMP-9 is thus reduced from the first trimester of pregnancy forward, the process of placental formation is disturbed, oxidative stress develops, and the clinical features of EO-PE ultimately occur. This sequence can explain the significant diminution in MMP-9 in the group of women with EO-PE compared to those with late-onset PE. This also confirms the hypothesis that EO severe PE is a true placenta-associated complication and that its development is due to defects in implantation and remodeling of the spiral arteries—a process in which MMP-9 is actively involved.

We found confirmation of our data in numerous studies. Coolman et al. revealed an elevated level of MMP-9 in uncomplicated pregnancies, as it provides complete remodeling and adequate angiogenesis during placental formation that is sufficient for normal growth of both the placenta and the fetus [12]. Su et al. [13] believe that an attenuation in the levels of MMP-2 and MMP-9 leads to disordered trophoblast invasion and that an increased level of MMP-2 causes endothelial damage and progressive vasoconstriction. However, our studies are not consistent with the results of Prochazka et al. [14], who reported no statistically significant differences in the levels of MMP-2 and MMP-9 in PE compared with uncomplicated pregnancies. In contradistinction, Poon et al. [15] demonstrated increased expression of MMP-9 during pregnancies complicated by PE.

The findings of our study confirmed the role of MMP-2 and MMP-9 in the genesis of severe PE. EO-PE and LO severe PE were associated with an augmented concentration of MMP-2 and a commensurately attenuated level of MMP-9. The low levels of MMP-9 reflect inadequate angiogenesis in the formation of the placenta, which in turn leads to placental ischemia and oxidative stress—reflecting the first stage of the development of PE.

## 5. Conclusions

We found a significantly higher concentration of MMP-2 in patients with clinical features of severe PE, confirming the participation of MMP-2 in the second stage of PE implementation, i.e., endothelial damage, development of arterial hypertension, and multiple-organ damage. The significantly elevated level of MMP-2 we obtained in pregnant women—both with EO-PE and LO severe PE—confirmed the development of endothelial dysfunction as an important element in the pathogenesis of polyorganic damage in PE.

The future direction of our research entails an assessment of the progression of MMP levels in preeclamptic patients and the calculation of cut-off levels of MMPs for predicting complications of PE and the prognosis of adverse maternal outcomes.

## Figures and Tables

**Table 1:** Clinical characteristics of EO-PE and LO-PE patients and control subjects.

Parameters	Severe EO-PE (*n* = 30)		Severe LO-PE (*n* = 31)		Control group (*n* = 31)
Age (years)	32.09 ± 6.48	*p* = 0.734311	31.78 ± 5.32	*p* = 0.73427	29.37 ± 4.65
Pregnancy	2.45 ± 1.63	*p* = 0.516627	2.35 ± 1.59	*p* = 0.54519	1.30 ± 0.67
Parity	1.60 ± 0.84	*p* = 0.704630	1.66 ± 0.88	*p* = 0.67238	1.2 ± 0.63
BMI (kg/m^2^)	31.89 ± 5.38	*p* = 0.532602	31.73 ± 5.76	*p* = 0.56055	27.14 ± 5.32
Systolic blood pressure (mmHg)	164.76 ± 21.18	*p* = 0.041171	161.39 ± 15.53	*p* = 0.01620	116.25 ± 9.54
Diastolic blood pressure (mmHg)	100.31 ± 10.9	*p* = 0.025082	98.65 ± 9.0	*p* = 0.01421	73.12 ± 4.58
Mean arterial pressure, MAPII (mmHg)	121.33 ± 10.9	*p* = 0.007691	118.46 ± 14.37	*p* = 0.05008	87.75 ± 5.39
Number of gestational weeks at birth	30.27 ± 2.90	*p* = 0.101560	37.07 ± 1.73	*p* = 0.81791	38 ± 3.63
Neonatal weight (g)	1526 ± 70.5	*p* = 0.032930	2840 ± 76.8	*p* = 0.65696	3210 ± 31.0
MMP-2 (ng/ml)	508 ± 137	*p* = 0.000393	491 ± 275	*p* = 0.02990	257 ± 123
MMP-9 (ng/ml)	1116 ± 389	*p* = 0.989952	834 ± 451	*p* = 0.84421	1391 ± 599

**Table 2:** MMP-2 and MMP-9 levels in the control group, EO-PE, and LO-PE.

Studied groups	MMP-2 (нг/мл)	MMP-9 (нг/мл)
Control group (*n* = 31)	257 ± 123	1391 ± 599
*p*, control vs. EO-PE	^∗^ *p* = 0.00039379	0.00884
EO-PE (*n* = 30)	508 ± 137	1116 ± 389
*p*, EO-PE vs. LO-PE	*p* = 0.93676	0.35274
LO-PE (*n* = 14)	491 ± 275	834 ± 451
*p*, control vs. LO-PE	^∗^ *p* = 0.029909	0.034222

^∗^Statistical significance at *р*˂0.05.

## Data Availability

The data used to support the findings of this study were supplied by the I.M. Sechenov First Moscow State Medical University Department of Obstetrics, Gynecology, and Perinatology and therefore cannot be made freely available. Requests for access to these data should be made to the corresponding author: Elena Timokhina (E-mail: elena.timokhina@mail.ru; Tel. +7916-6074534).

## Abbreviations

MMP: Matrix metalloproteinases
PE: Preeclampsia
EO-PE: Early-onset preeclampsia
LO-PE: Late-onset preeclampsia
AH: Arterial hypertension
BP: Blood pressure
SBP: Systolic blood pressure
DBP: Diastolic blood pressure
CNS: Central nervous system
HELLP syndrome: Hemolysis, elevated liver enzymes, thrombocytopenia
IGR: Intrauterine growth retardation
BMI: Body mass index.
